# Identifying Solutions for the Workforce Challenges Facing Community Mental Health Support Workers: A Qualitative Study

**DOI:** 10.1007/s10597-025-01473-w

**Published:** 2025-05-22

**Authors:** Megan Rattray, Emma Milanese, Tania Shelby-James

**Affiliations:** Discipline of General Practice, College of Medicine & Public Health, Flinders University, Adelaide, South Australia Australia

**Keywords:** Psychosocial, Workforce, Recruitment, Retention, Skill, Mental health

## Abstract

Addressing workforce issues such as recruitment, retention, and skill development is essential for ensuring that community psychosocial and mental health programs can meet growing demand and provide high-quality care. This paper examines current efforts to tackle these challenges and explores additional measures required. This qualitative study, framed within a naturalistic paradigm, employed semi-structured interviews with mental health support staff across Australia. Interviews, lasting approximately 37 min (range, 23–55 min), were audio recorded and transcribed verbatim. Data were analysed using a combination of inductive and deductive thematic analysis, whereby participants’ verbatim codes were initially mapped across the three workforce issues: recruitment, retention, and skill development. Solutions for each issue were then analysed inductively, with subthemes emerging from common concepts. Themes and subthemes were discussed among the authors until consensus was reached. Sixteen staff members were interviewed, most of whom were female (*n* = 9, 56%) and held management roles (*n* = 10, 62%), while the rest were in client-facing positions (*n* = 6, 38%). To improve staff recruitment, subthemes included revising recruitment processes and standards, dispelling misconceptions and promoting the field and leveraging existing networks and resources. Staff retention subthemes included offering competitive salary packages and incentives, fostering a positive work culture with engaged leadership and encouraging supportive and effective practices. Lastly, upskilling staff subthemes included engaging in regular or mandatory training and supervision, encouraging resource sharing and networking and supporting and granting opportunities to upskill. The findings offer insights and recommendations for policymakers, administrators, and practitioners to strengthen the community mental health workforce.

**Supplementary Information:**

The online version contains supplementary material available at 10.1007/s10597-025-01473-w.

## Background

Mental ill-health is a global issue, profoundly impacting individuals and societies worldwide (Ngui et al., 2010; Wainberg et al., 2017). In Australia, an estimated 1 in 5 adults will experience a mental disorder in any given year, with nearly half the population expected to experience mental ill-health at some point in their lives (Australian Institute of Health and Welfare, 2024). Among the services available, the Commonwealth Psychosocial Support Program (CPSP) provides tailored support to over 18,000 individuals living with mental illness. This program is designed to enhance daily functioning, support personal recovery goals, and improve overall quality of life (Australian Government, 2023).

The quality of programs like the CPSP are closely tied to the capabilities of the mental health workforce. Within the CPSP, services are delivered by community-based mental health organisations which are predominantly from the not-for-profit sector. The workforce delivering these services primarily consist of mental health support workers, who occupy direct care, management, and administrative roles. Further, a notable portion of this workforce includes Lived Experience (LE) workers - individuals who have personally navigated mental ill-health and recovery (National Mental Health Commission, 2024). These staff bring invaluable insight, empathy, and understanding to their roles, enriching the care provided (National Mental Health Commission, 2024). Importantly, this workforce is predominantly vocationally trained (VET sector), often holding qualifications such as a Certificate IV in Mental Health, rather than university degrees.

Community-based organisations form a key pillar of Australia’s mental health service system, operating alongside public and private sector providers. However, recent reports and studies have highlighted significant workforce challenges within this sector, particularly around staff recruitment, retention, and skill development (Australian Government, 2022; Cosgrave et al., 2015; Cosgrave et al., 2018; Queensland Alliance for Mental Health, 2023; Rattray, 2024). A recent mixed-method national study conducted by the current authors highlighted the complexity and multifaceted nature of these workforce issues (Rattray, 2024). Specifically, poor recruitment was linked to an interplay of factors including short-term contracts, a lack of specialisation in mental health within undergraduate programs and limited awareness of mental health in general, inadequate salary packaging, and negative perceptions of the workforce (Rattray, 2024). Poor retention was associated with factors such as burnout, job insecurity, limited career opportunities, poor workplace culture, and job dissatisfaction (Rattray, 2024). Lastly, inadequate skill development was attributed to infrequent professional development, insufficient on-the-job training, limited mentoring and supervision, and unclear role expectations (Rattray, 2024). Addressing these workforce challenges is crucial to ensuring that community mental health programs can meet growing demand and provide high-quality care.

Limited research has been conducted to explore solutions to these three overarching issues. While several reports have proposed solutions (Australian Government, 2022; Queensland Alliance for Mental Health, 2023), no comprehensive peer-reviewed work has been published that thoroughly explores solutions to these challenges using comprehensive qualitative methods. Qualitative methods allow for a deep exploration of the experiences, perspectives, and insights of those directly involved in the mental health workforce (Renjith et al., 2021). Therefore, the aim of this paper is to build upon our previous work by exploring mental health support staff’s perspectives of current organisational efforts to address issues related to recruitment, retention, and skill development, and to identify additional measures that could be implemented. These participants were drawn from the same broader workforce sample as the earlier study, providing continuity and depth to the investigation. By identifying and analysing successful strategies and potential improvements, this study seeks to contribute to the development of a more robust and capable workforce in the community psychosocial and mental health sector.

## Methods

This qualitative study was suited in a naturalistic paradigm (Lincoln & Guba, 1985) using semi-structured interviews with Australian mental health support staff to examine current and desired initiatives addressing workforce issues, including recruitment, retention, and skill development. Ethical approval was obtained from the Flinders University Human Research Ethics Committee (Project ID: 5675). The study’s methodology and reporting were aligned with the Consolidated Criteria for Reporting Qualitative Research (COREQ) guidelines (Tong et al., 2007) and the Standards for Reporting Qualitative Research (SRQR) guidelines (O’Brien et al., 2014).

### Study Context

Under the CPSP, the Australian Government provides funding to 31 Primary Health Networks (PHNs) to commission psychosocial support services for people with severe mental illness and associated psychosocial functional impairment who are not accessing similar supports through the National Disability Insurance Scheme or state and territory-based programs. These service providers are community-based mental health organisations that may serve an entire PHN region or a more localised area.

### Recruitment

Staff were recruited through a national survey examining mental health support workers’ perceptions of factors affecting recruitment, retention, and skill development. Eligible participants included part- or full-time staff members employed by a PHN or one of their commissioned service providers. The survey was distributed to all 31 PHNs and their commissioned psychosocial service providers across Australia. At the end of the survey, participants were asked if they were interested in being contacted for a semi-structured interview to discuss potential solutions to these issues, with a brief explanation of the interview process. Among those who expressed interest, an information sheet was emailed by a study team member (MR or EM), and interview times were scheduled at mutually convenient times. Verbal consent was reconfirmed at the beginning of each interview. Participants were purposefully selected to ensure a diverse representation based on georgical location, work setting and role type. While it was initially estimated that 15–20 interviews would be conducted, recruitment continued until data saturation was reached, that is, when no new themes or concepts emerged in relation to recruitment, retention, or skill development (Denzin & Lincoln, 2011). Saturation was observed after the fourteenth interview, however, two additional interviews were conducted to confirm that no new information emerged.

### Data Collection

The interviews were conducted between October and December 2023. Using a semi-structured interview guide developed by the authors, participants’ perceptions were explored through a series of broad questions related to the three key issues: recruitment, retention, and skill development. Specifically, participants were asked, “What is your organisation currently doing to address these issues?”, “What more would you like to see your organisation do to address these issues?”, “Who else besides your organisation is responsible for addressing these issues?”, and “What would you like to see these organisations do?”. A conversational interviewing approach was employed, allowing the interview guide and participant responses to steer the discussion. Two female authors (MR and EM), both experienced interviewers, conducted the one-on-one interviews via telephone or Microsoft Teams. Some participants were already familiar with the interviewers, as they are part of a team providing capacity-building supports for the CPSP. The average interview duration was 37 min (range: 23 to 55 min). All interviews were audio recorded and were transcribed verbatim using Otter.ai, an artificial intelligence-based real-time transcription service (Lau, 2024), and were subsequently cross-checked for accuracy. Participants did not have the opportunity to review their transcripts.

### Data Analysis

The interview data was managed in Microsoft Word. Thematic analysis was employed to examine the textual data from the interviews. Initially, deductive thematic analysis was conducted in which codes directly derived from verbatim statements were mapped across the three issues by the lead author. Subsequently, codes related to solutions for each issue were analysed inductively which allowed subthemes based on common concepts to emerge. This combination of deductive and inductive approaches provides a systematic and objective method for making valid inferences and quantifying phenomena (Elo & Kyngäs, 2008). The labelling of themes and subthemes was discussed among all authors until consensus was reached. Throughout the process, Braun and Clarke’s six-step guide to thematic analysis was closely followed to ensure a rigorous and structured approach (Braun & and Clarke, 2006).

Several strategies outlined by Lincoln and Guba (Lincoln & Guba, 1985) were employed to maximise trustworthiness and validity of our findings. For example, we present detailed and in-depth descriptive data and include rich verbatim quotes from participants that depict each theme to enhance transferability and confirmability of our findings. Further, we enhanced credibility and dependability through using open-ended questioning, spending a prolonged amount of time listening to and reading the transcripts and giving a detailed description of the methods and direct quotes from participants.

## Results

Fifty-nine participants indicated their interest in participating in an interview. From this pool, 36 individuals were extended invitations, and ultimately, 16 participants were interviewed. A breakdown of the demographics for the interviewed participants is presented in Table 1. Participant responses formed three themes and various subthemes, described in detail below.

**Table 1:** Interviewed participant demographics (*n* = 16)

Demographics		*N* (%)
Work setting		
	Primary Health Network	2 (13%)
	Service Provider	14 (87%)
Employment status		
	Full time	11 (69%)
	Part time	5 (31%)
Position		
	Management type role	9 (56%)
	Client support role	6 (38%)
State / Territory		
	South Australia	4 (25%)
	Tasmania	1 (6%)
	Queensland	1 (6%)
	Vicotria	5 (31%)
	New South Wales	3 (19%)
	Northen Territory	0 (0%)
	ACT	0 (0%)
	Western Australia	2 (13%)
Geographical location		
	Metropolitan	5 (31%)
	Regional	7 (44%)
	Rural or remote	4 (25%)
Current age		
	30–34	2 (13%)
	35–39	1 (6%)
	40–44	4 (25%)
	45–49	2 (13%)
	50–54	1 (6%)
	55–59	1 (6%)
	60–64	5 (31%)

### Theme 1: Addressing Recruitment Challenges

Interviewed participants discussed a range of strategies that their workplaces are currently employing to address recruitment challenges, as well as additional approaches they would like to see implemented both within their organisation and/or by others.

#### Revising Recruitment Processes and Standards

To improve staff recruitment, several staff shared examples of processes or standards their workplaces have modified or could consider. One participant in a management role described that their workplace had established a working party to develop an Employee Value Proposition. This involved exploring how current employees feel valued, identifying areas for improvement, and incorporating these insights into recruitment efforts to enhance job desirability. Another participant in a management role recounted how, after a “*bad round of recruitment*,” their organisation adjusted its interview process. They provided clearer role information in job ads and included relevant resources, increasing role transparency and allowing candidates to better prepare for interviews:

“Previously, we’ve gone into recruitment as a bit of a test… like, ‘Okay, let’s see what you can bring to the table. And I’m not going to give you the answers you, you have to kind of read my mind’… Whereas this time, we went into it with a lot more transparency, and like, part of our job advertisement had links to websites and things like that, so that people could actually… be prepared with their application and be prepared for their interview… and I had conversations with people like… ‘is there any other clarity that you need around what this role could entail that’s going to prepare you?’… so trying to be a little bit more proactive in that space and not testing them so much to already know the answers coming into it.” [P03: management role at a metropolitan service provider].

Additionally, modifying recruitment standards was discussed as a potential solution; however, this received diverse views. Some staff members emphasised the importance of clearly specifying qualification standards in job advertisements, such as requiring a Certificate IV in Mental Health, to attract the “*right*” candidates, given the increasing complexity seen in the CPSP. In contrast, others spoke about lowering qualification standards in attempt to attract more applicants, with the idea of that individuals can be upskilled once they were in the position.

#### Dispelling Misconceptions and Promoting the Field

Many participants provided suggestions on how misconceptions about mental health could be demystified and the field made more appealing to recruit candidates. They highlighted the importance of universities and Technical and Further Education (TAFE) centres focusing more on mental health education to portray it as a rewarding career path with diverse opportunities. Suggestions included providing real-world experiences and practical training to inform and adequately prepare individuals for the mental health work. Hosting traineeships and student placements was also discussed. For example, peer candidates were viewed as a promising avenue, with suggestions to collaborate with TAFE and/or across organisations to enhance industry-specific traineeships, attracting individuals interested in upskilling while simultaneously delivering the CPSP. Another approach mentioned was hosting students, with one staff member sharing that their workplace regularly hosts social work students, which often leads to the recruitment of some graduates. In fact, one of the interviewed participants shared that they acquired their current job after completing a placement at their current organisation. Lastly, there were suggestions that mainstream conversations about mental health needed to shift to increase the desirability of working in the field. This was described as upskilling the public’s understanding of disability and psychosocial support and normalising mental health awareness, with one staff member suggesting that this comprehensive information dissemination needs to be done by the Australian Government.

“There needs to be a stronger focus in universities and TAFE around mental health… [for example] how can we make it a more attractive sector for people to want to work in…we need to be really driving home that, you know, mental health is a really rewarding career and, you know, there are lots of opportunities… you can work with young people, you can work with elderly people, you can just work with adults, you can work in clinical work and work in communities… you’re not stuck in a box. I think we need to sell that a little bit more.” [P05: management role at a regional PHN].

#### Leveraging Existing Networks and Resources

Several staff members shared how their workplaces effectively utilise available networks and resources to recruit staff, while others offered suggestions on how this could be achieved. For instance, one participant in a management role at a rural and remote service provider shared that they facilitated recruitment by hosting a free training session for individuals entering the aged care and disability sectors on behalf of the Australian Medical Association. By providing attendees with employee packs for their organisation, this initiative effectively engaged potential recruits. Similarly, another rural staff member shared their efforts to identify internal talent within their organisation who possessed skills relevant to supporting individuals with complex mental health issues and approached these workers to apply for roles within the CPSP:

“We’ve identified people who work in other areas of the organisation… we have employment consultants, and we also have disability support workers. I speak to the managers about anybody who might have some expertise in supporting people with complex mental health issues. Sometimes we can maybe utilise their input for a short time, or even encourage them to apply for a role if it comes up. One of our team members did use to work for the Disability Employment Services, and now she’s enjoying the role with us.” [P10: management role at a rural and remote service provider].

This same staff member then proposed the idea of collaborating across organisations to train and recruit staff who could move between the organisations as needed. Additionally, a different staff member suggested diversifying recruitment efforts by targeting professionals from nontraditional sectors such as construction or hospitality, with the aim to attract individuals willing to upskill and transition into mental health roles, leveraging their transferable skills and resilience. Lastly, the importance of developing and supporting a LE workforce was highlighted as a way to enhance recruitment by including individuals with firsthand mental health experience. While service providers may already be pursuing this approach, it was suggested that the Australian Government should mandate the inclusion of LE roles not only in service providers but also in PHNs. It was also noted that careful consideration must be given to training and supervision, ensuring these individuals are well-supported and adequately trained once in their positions as one staff member shared that three LE workers in their organisation left their positions shortly after employment, an outcome which was attributed to them feeling “*triggered*”.

### Theme 2: Enhancing Staff Retention

Staff discussed current strategies their workplace uses to address the issue of retaining staff and additional approaches they wish to see implemented.

#### Offering Competitive Salary Packages and Incentives

The concept of offering fair salary packaging and incentives was discussed in both recruiting and retaining staff, with a greater emphasis on staff retention. While some staff mentioned paying their employees competitively to recruit and retain them, and a few employees expressed satisfaction with their current salary, the idea of standardising pay across organisations and outright paying staff more was acknowledged as a potential strategy to address recruitment and retention challenges by a number of staff. However, some participants noted that increasing pay needs to be an industry-wide initiative, as many organisations are constrained by current award rates. The idea of offering incentives beyond pay was also discussed. Some incentives that staff believed would help with recruitment and retention, included covering moving expenses and allowing staff to choose between an extra week of leave per year or opting for a wellbeing hour once a week for activities like yoga or walking. Some staff did share that the latter strategy is currently embedded in their Enterprise Bargaining Agreement (EBA):

“We’ve done a lot of work around our employee value proposition. People can opt in to having a… wellbeing hour, once a week. So they can do a yoga class or have a walk or do whatever, or they can trade that and have a 5th week of leave.… people mostly prefer time rather than more money. Although we did put everyone up a band a couple years ago. So making sure that there’s a good working culture and people can have access to some staff benefits that support wellbeing is something that we’ve done.” [P08: management role at a metropolitan service provider].

The Employee Assistance Program (EAP) was also discussed, which covers a few free sessions with a psychologist. However, one staff member noted that this was not seen as a sufficient incentive to stay.

#### Fostering a Positive Work Culture with Engaged Leadership

Cultivating a positive work culture with interactive leadership was another key concept discussed by staff for retaining employees. Given financial constraints limiting pay raises, staff emphasised the importance of feeling valued and safe in their workplace. They highlighted the need for continuous efforts to ensure communication and transparency between management and employees. Some staff shared how their workplace is currently achieving this, while others discussed potential methods to improve this. Collectively, this was described as management fostering open communication through regular formal and informal check-ins to address challenges collaboratively, investing in leadership training, being honest and open, conducting workplace cultural reviews, setting clear vision and goals, and promoting inclusivity.

“The only way you can retain staff is having an exceptionally good culture within your organisation… a good culture is one where people are very, very, very honest… [where] we [can] challenge what’s going on… but where we also support individuals [and when that’s the case] people feel that they are looked after, and that they are safe in their own workplace.” [P12: management role at a rural and remote service provider].

#### Encouraging Supportive and Effective Practices

Promoting good practices and offering support was another strategy emphasised by staff to retain employees and prevent burnout. Some staff shared current practices in their organisation to mitigate burnout, while others discussed desired improvements. From a managerial perspective, this involved allowing flexible work arrangements, accommodating employee leave requests, and providing regular formal or informal mentoring and supervision. One participant in a management position mentioned negotiating with funders in the past to ensure manageable caseloads for their staff. From a frontline worker’s viewpoint, this included implementing practices to reduce administrative burdens, such as collaborating with clients on tasks like referrals and finding convenient locations to complete case notes during extended driving periods. Additionally, two frontline workers highlighted that their team is supportive of each other, which helps prevent caseloads from becoming overwhelming. A participant in a managerial role echoed this sentiment sharing that they encourage the practice of having at least two staff members aware of each client in the CPSP to reduce burnout as this approach ensures that multiple perspectives are available for handling challenging situations. However, they acknowledged that maintaining this arrangement is not always feasible. This same participant then shared how they’re currently “*encouraging team members to build alliances with other agencies”* to *“informally case manage clients across two or three agencies*.” [P10: management role at a rural and remote service provider].

### Theme 3: Strengthening Staff Skills

Staff discussed strategies that their workplace either currently implements or could implement to upskill staff.

#### Engaging in Regular or Mandatory Training and Supervision

Regarding engaging in regular or mandatory training and supervision, staff mostly shared positive examples of how their organisation already excels in this area. For instance, some staff mentioned that their organisation hosts mandatory professional development sessions every 2–3 months, covering topics such as alcohol and drug awareness, working with youth, and supporting LGBTQIA + communities. One participant from a PHN shared that they previously saw it as the responsibility of their commissioned service providers to upskill their staff. However, upon reflection, they realised that their service providers need support to achieve this:

“I’ve got a procurement plan actually going to the board tomorrow… to build in funding within our schedule for… regularly offering like family domestic violence training, trauma informed care and practice, AOD stuff… because I just really think initially, we thought, well, that is on the organisations that employ the staff, but we know that they’re very limited in their resources. So we’re trying to support that as well. But you know, it’s an ongoing thing, because as staff are turning over, there’s new people coming in, then you kind of need to be offering that on a regular basis.” [P05: management role at a regional PHN].

Additionally, one staff member mentioned their organisation’s practice of providing new recruits with a probation document outlining required training in their first 5 months, along with a personalised development plan called ‘grow’ for the year ahead, were useful tools to assist employees in upskilling. In terms of mentoring and supervision, two staff members described the formalised process in their organisation, where they have scheduled monthly one-hour meetings with their manager, which they find beneficial.

#### Supporting and Granting Opportunities To Upskill

Supporting and providing opportunities for staff to upskill was another aspect highlighted positively by staff regarding their organisation’s efforts. For example, some staff mentioned how their organisation encourages a culture where employees can take charge of their own learning. Within this, staff shared that they identify the training they need and discuss its relevance with management. If there are associated costs, they can then collaborate to see if it fits within the budget. Staff who discussed this process noted that permission is usually granted in such cases. However, one participant in a management role shared that they would like to see their organisation be more transparent with the training budget allocated for each person and that at times a more proactive approach us required in following up with employees about their training needs:

“I think we just have… [to] make sure that we keep working with people around what they’d like to do and being a bit more assertive about that. Like ‘you said you wanted to do this last month, you know, have you looked into that? Can I help you to find that?’… With some people, they’re very self-directed, but with others, they maybe need a little bit more of a nudge. So I guess it’s just identifying that and following through on those actions.” [P01: management role at a metropolitan service provider].

Additionally, some staff mentioned how their workplace supports employees in completing their Certificate IV in Mental Health by allowing them the necessary time to pursue it.

#### Encouraging Resource Sharing and Networking

Encouraging resource sharing and networking was another proposed solution mentioned by some staff, with these staff primarily highlighting effective practices already in place within their organisations. For instance, some staff mentioned that their teams actively share useful free online trainings, supported by management’s promotion of ongoing training opportunities like webinars. Regarding networking, one staff member described how their community of practice (CoP) facilitates mutual learning, while another mentioned that these sessions had previously paused due to resource constraints but are now restarting, as they see the potential of these sessions in promoting networking, reflection, and reducing isolation among staff. However, the staff member who mentioned that they are already running quarterly face-to-face CoP spoke about the following drawbacks:

“I do like to do most of my community of practices face to face. That’s challenging as it is costly for us as an organisation, because… our region is massive. So trying to get everybody together…. We’ve found that specifically for the community practice, not so much the education sessions, but with a community of practice, we found to get buy in we had to pay for them to begin with. Like to show them that it’s worth coming… it’s hard to get across the line, you know, you’re taking a big chunk out of operational expenses to do that, which we don’t get much off from the Department anymore… We’ve got to spend money out of our operational fees to get these organisations together, so they can deliver the best service for the community.” [P06: management role at a regional PHN].

## Discussion

This study explored the initiatives currently being undertaken to address workforce challenges in recruitment, retention, and skill development, while also considering additional measures that staff believe could have an impact. Many of these solutions were framed within the context of what service providers are presently implementing or what employees wish to see their organisations address. Nevertheless, some solutions for enhancing recruitment and retention extended to the roles that PHNs, the Australian Government, and educational institutions (e.g., universities and TAFEs) could implement. Given the recent release of the National Mental Health Workforce (NMHW) Strategy 2022–2032 (Australian Government, 2022) and the Community Mental Health and Wellbeing Workforce (CMHWW) Issues Paper (Queensland Alliance for Mental Health, 2023), the solutions identified in this study for recruitment (Supplementary Material 1), retention (Supplementary Material 2) and staff skill (Supplementary Material 3) will be contextualised in relation to these reports.

To enhance recruitment, staff in the current study proposed revising recruitment processes and standards, dispelling misconceptions and promoting the field, and leveraging existing networks and resources. Collectively, these strategies address some of the factors reported for poor staff recruitment such as limited pool of applicants, lack of specialisation in mental health undergraduate programs and limited awareness and negative perceptions of the workforce (Rattray, 2024). Specifically, participants noted that strategies such as developing an Employee Value Proposition and improving role transparency have proven effective in attracting suitable candidates. These feasible, immediately implementable approaches align with best practices that emphasise clear communication and alignment between job roles and candidate expectations (Gara & La Porte, 2020) and thus help address the issue of limited awareness among the general public.

Additionally, staff mentioned the importance of improving the public’s perception of mental health, addressing stigma, and collaborating with educational institutions to implement recruitment pathways—recommendations also highlighted in the NMHW Strategy 2022–2032 (Australian Government, 2022) and the CMHWW Issues Paper (Queensland Alliance for Mental Health, 2023). While initiatives like student placements and industry collaborations have proven effective in other sectors (Fleming, 2023) and would help address negative perceptions of the workforce, they may require years to fully implement. In the interim, resourceful approaches, such as engaging potential recruits through opportunistic training sessions, identifying internal talent for complex mental health roles, collaborating locally to train and recruit for flexible roles, and targeting professionals from nontraditional sectors like construction or hospitality, were suggested to expand the recruitment pool. The CMHWW Issues Paper (Queensland Alliance for Mental Health, 2023) advocates for promoting career-change pathways, particularly for Aboriginal and Torres Strait Islander peoples, culturally and linguistically diverse (CALD) communities, and the LGBTIQ + population. It also recommends developing a tool to support these efforts and encourages greater local collaboration through workforce initiatives. These strategies would not only broaden the recruitment base but also foster diversity and inclusion within the mental health workforce, which is essential for responding to the varied needs of the community (Australian Government, 2022).

Lastly, both the NMHW Strategy 2022–2032 (Australian Government, 2022) and the CMHWW Issues Paper (Queensland Alliance for Mental Health, 2023) emphasise the crucial role of LE workers and outline practical steps for their successful integration. While this was identified as a solution in the current study, staff highlighted the challenges of implementing this strategy due to the lack of a supportive framework and practices in the current environment and the need for balancing experience with qualifications. This highlights the need for more robust structures to effectively integrate LE workers and enhance the overall capability of the LE workforce (Byrne, 2021).Strengthening the integration of LE workers, supported by structured frameworks and organisational commitment, should be considered a particularly promising strategy for community-based services seeking to build a responsive and recovery-oriented workforce.

To improve retention, key strategies identified by staff in this study include offering competitive salary packages and incentives, fostering a positive work culture through engaged leadership, and encouraging supportive and effective practices. These findings echo previous research, which highlights sufficient funding as a top priority for employees in mental health roles (Mental health coordinating council, 2023) and identifies job insecurity and inadequate salary packaging as significant factors contributing to poor staff retention and recruitment (Rattray, 2024). It is therefore unsurprising that both the NMHW Strategy 2022–2032 (Australian Government, 2022) and the CMHWW Issues Paper (Queensland Alliance for Mental Health, 2023) also emphasise the need for competitive salaries and longer minimum service contracts to enhance job security. In the current constrained funding environment of community-based organisations, advocating for competitive salary packages through funders, alongside strategies that build intrinsic workplace value (e.g., supportive leadership and flexible working conditions), are likely among the most realistic and impactful retention strategies.

In terms of fostering a positive work culture with engaged leadership—also highlighted in both the national and state reports (Australian Government, 2022)—staff suggested creating a safe, inclusive environment, maintaining open communication, investing in leadership training, conducting cultural reviews, setting clear goals, and regularly checking in with employees. These suggestions align with research highlighting that effective leadership practices, such as open communication and inclusivity, have been shown to boost employee morale and reduce turnover (Coates & Howe, 2015). Staff also proposed several strategies to promote supportive and effective practices, including flexible work arrangements, regular mentoring and supervision, negotiating manageable caseloads with funders, streamlining work processes, fostering team support to share responsibilities, and building alliances with other agencies to distribute workload. While the NMHW Strategy 2022–2032 (Australian Government, 2022) and the CMHWW Issues Paper (Queensland Alliance for Mental Health, 2023) also advocate for increased access to supervision and mentoring, they tend to focus more on broad solutions and strategic initiatives, such as clearly defined career pathways and professional development, rather than the day-to-day solutions to improve employee wellbeing proposed in the current study. This highlights a key opportunity for community-based organisations to implement low-cost, relational strategies, such as improving supervision quality and investing in leadership development as “best bets” for addressing burnout and improving retention under resource-constrained conditions.

To strengthen staff skills, participants in the current study emphasised the importance of engaging in regular or mandatory training and supervision, encouraging resource sharing and networking, and supporting and granting opportunities to upskill. These priorities align with the NMHW Strategy 2022–2032 (Australian Government, 2022) and the CMHWW Issues Paper (Queensland Alliance for Mental Health, 2023), both of which highlight training and supervision as critical strategies for building workforce capacity; a common strategy used to increase knowledge and skill (Coates & Howe, 2015). Specifically, staff within the study identified the need for mandatory training sessions, structured probation plans, and formalised supervision meetings. The NMHW Strategy 2022–2032 (Australian Government, 2022) broadens this approach by advocating for enhanced training pathways, competency-building modules, and improved placement quality, while the CMHWW Issues Paper (Queensland Alliance for Mental Health, 2023) proposes the development of a core capability framework, micro-credentials, and coordinated learning initiatives such as a centralised professional development repository. Previous work also suggests that mental health workers support contracts that explicitly embed workforce capacity building elements, including training, supervision, mentoring, and resource provision (Australian Government, 2022). Although there is strong consensus on the need for regular training and professional development - particularly in light of the increasing complexity of client needs (Munasinghe et al., 2022; Rattray, 2024)– effectively implementing these strategies remains challenging, especially in rural and remote areas (Rattray, 2024). Recognising these barriers, staff in the current study proposed practical solutions such as increasing training budget transparency, fostering self-directed learning, sharing training costs, and promoting access to free resources, and establishing local CoPs. Consistent with previous research, initiatives that facilitate resource sharing, networking, and structured self-directed learning have been shown to foster mutual support, continuous improvement, and employee empowerment (Collin et al., 2012). Given the financial and logistical constraints often faced by community-based organisations, prioritising accessible, low-cost strategies such as these represents a particularly promising and practical approach to strengthening workforce skills.

### Limitations

Since this study was conducted exclusively in Australia, the findings may not be fully transferable to other countries with differing healthcare systems. To improve the transferability of the results, a comprehensive description of the participants’ workplace demographics are provided and we included individuals from a variety of geographical locations, such as metropolitan, regional, and rural or remote areas. However, the relatively small sample size of 16 staff members may have limited the inclusion of certain viewpoints. To mitigate this, purposive sampling was employed to capture a broad range of diversity in terms of gender, geographical location and role type. Despite these efforts, additional limitations commonly associated with qualitative research, such as the potential for researcher bias during data analysis and challenges in generalising findings to larger populations, must also be acknowledged.

## Conclusion

This study highlights that multifaceted approaches are required to address workforce challenges in the mental health sector. While current efforts show promise, there is a need for ongoing innovation and collaboration to enhance recruitment, retention, and skill development among mental health support staff. By adopting a combination of strategies discussed in this paper, organisations can better support their staff and improve the quality and sustainability of mental health services. Further research and policy initiatives should focus on refining and implementing these strategies and exploring new avenues to strengthen the mental health workforce.

## Electronic supplementary material

Below is the link to the electronic supplementary material.

Supplementary Material 1

Supplementary Material 2

Supplementary Material 3
