# Supplementary material for: Identifying Solutions for the Workforce Challenges Facing Community Mental Health Support Workers: A Qualitative Study
Source: Community Ment Health J. 2025 May 22;61(7):1324–33. doi: 10.1007/s10597-025-01473-w (PMC12408665; doi:10.1007/s10597-025-01473-w)
Supplement: Supplementary file 1 — Supplementary Material 1 [file 10597_2025_1473_MOESM1_ESM.docx]

| **Supplementary Material 1.** Summary and comparison of proposed solutions for improving **recruitment**: insights from this study and two recent Australian workforce documents | | |
| --- | --- | --- |
| **Study generated solutions** | **National Mental Health Workforce Strategy 2022-2032 generated solutions*** | **Community Mental Health and Wellbeing Workforce Issues Paper*** |
| **Revising recruitment processes and standards (subtheme 1.1)**  •Create an Employee Value Proposition by assessing how employees feel valued and using this information to improve job roles and attract recruits.  •Revise interview process by providing clearer role information and relevant resources to improve candidate preparation.  •Maintain high qualification requirements to attract well-qualified candidates or lower them to broaden the applicant pool and upskill new hires.  **Dispelling misconceptions and promoting the field (subtheme 1.2)**  •Universities and TAFEs to enhance MH education by highlighting it as a rewarding career path and providing real-world experiences and practical training.  •Develop industry-specific traineeships with TAFEs and hosting student placements, with some graduates securing jobs after such placements.  •Shift mainstream conversations about mental health to enhance its appeal as a career by improving public understanding, normalising awareness, and providing comprehensive information, potentially led by the Government.  **Leveraging existing networks and resources (subtheme 1.3)**  •Capitalise on opportunities to engage potential recruits e.g. through volunteering to host free training sessions for staff entering relevant sectors.  •Identify internal talent with relevant skills for complex MH issues and encourage them to apply for CPSP roles.  •Collaborate with local agencies to train and recruit staff for flexible roles. •Target professionals from nontraditional sectors, like construction or hospitality, to attract. •Develop and support a LE workforce with consideration given to training, supervision. | **Develop and deliver recruitment and career pathways to attract a suitably skilled and diverse workforce (strategy 1.4)**  •Collaborate with the education sector to strengthen recruitment pathways (1.4.1).  •Recruit people from regional, rural/remote communities to complete MH education (1.4.2).  •Strengthen recruitment and integration support pathways for overseas workers (1.4.3).  •Develop and implement career pathways within and between MH and health service settings (1.4.4).  **Promote mental health careers as an attractive career choice (strategy 1.3)**  •Raise the awareness of pathways into, and within, the workforce for both vocational and higher education trained occupations (1.3.1).  •Address stigma and negative perceptions associated with working in MH (1.3.1).  •Create positive perceptions of working in MH by improving the pre-service/ placement experience of students/trainees (1.3.1).  **Address critical shortages in the mental health workforce (strategy 1.1)**  •Examine innovative service delivery models to support increased engagement of the LE and First Nations workforces in different contexts (1.1.2).  •Establish educator roles to support the further development of the LE and First Nations MH workforce (1.1.3).  •Collaborate with consumer, carer and LE worker representatives to support establishment of a national LE workforce peak organisation (1.1.4).  •Create incentives for employers to increase the numbers of designated LE employees across all organisational levels (1.1.6). | **Geographic distribution (pillar 2.2)**  •Fund traineeships for the Certificate IV qualifications under the User Choice Program so that communities can ‘grow their own’ workforces.  **Increase supply (pillar 2.1)**  •Develop a state-wide campaign to increase awareness of the value of the mental health sector.  •Enhance school-to-work transitions by creating clear pathways for young people  leaving school.  •Enhance/promote change-of-career pathways, with consideration given to targeting specific populations (Aboriginal and Torres Strait Islander peoples, CALD and LGBTIQ+).  •Develop an online tool for potential workers to self-assess suitability and the skills required to work in the sector.  •Work with state to address stigma and discrimination facing the mental health sector.  **Integration (pillar 3.3)**  •Support increased local collaboration through the development of workforce initiatives e.g. shared recruitment processes, induction training, secondments, exchange programs, sabbaticals, coaching, mentoring and reflective practices.  **LE Workforce (pillar 3.4)**  •Develop traineeships or ‘Peer Work Internships’ that align with TAFE qualifications.  •Develop a training package / microcredential for LE workers to be delivered on induction. •Ensure appropriate HR policies and procedures are in place to support the wellbeing of LE workers, including access to peer supervision and mentoring.  •LE Workforce strategies continued…. |
| *Wording of some strategies has been adjusted to convey more concisely.  CALD: Culturally and Linguistically Diverse; CPSP: Commonwealth Psychosocial Support Program; LGBTIQ+: Lesbian, Gay, Bisexual, Transgender, Intersex, and Queer/Questioning; MH: Mental health; LE: Lived Experience; TAFE: Technical and Further Education. | | |
