# Supplementary material for: Identifying Solutions for the Workforce Challenges Facing Community Mental Health Support Workers: A Qualitative Study
Source: Community Ment Health J. 2025 May 22;61(7):1324–33. doi: 10.1007/s10597-025-01473-w (PMC12408665; doi:10.1007/s10597-025-01473-w)
Supplement: Supplementary file 2 — Supplementary Material 2 [file 10597_2025_1473_MOESM2_ESM.docx]

| **Supplementary Material 2**. Summary and comparison of proposed solutions for improving **retention**: insights from this study and two recent Australian workforce documents | | |
| --- | --- | --- |
| **Study generated solutions** | **National Mental Health Workforce Strategy 2022-2032 generated solutions*** | **Community Mental Health and Wellbeing Workforce Issues Paper*** |
| **Offering competitive salary packages and incentives (subtheme 2.1)**  •Advocate for industry-wide salary adjustments to standardise and offer competitive pay.  •Offer incentives beyond pay such as covering moving costs, allowing employees to choose between an extra week of annual leave or a weekly wellbeing hour for activities like yoga, and ensuring they are aware of free psychological support sessions available to them.  **Fostering a positive work culture with engaged leadership (subtheme 2.2)**  •Create a work environment where employees feel valued and safe.  •Engage in continuous efforts to maintain open communication and transparency between management and employees.  •Engages in regular check-ins, invest in leadership training, be honest and open with employees, conduct cultural reviews, set clear vision and goals, and promote inclusivity.  **Encouraging supportive and effective practices (2.3 subtheme)**  •Offer flexible work arrangements and accommodating employee leave requests.  •Provide regular formal or informal mentoring and supervision.  •Management negotiating with funders to ensure staff have manageable caseloads.  •Front line workers to complete tasks with clients (e.g. referrals) and finding convenient locations to complete case notes during extended driving periods.  •Encourage team support to manage caseloads, as it allows for shared responsibilities and reduces individual stress. •Team members to build alliances with other agencies and informally case manage clients across multiple agencies to distribute workload. | **Adopt funding models and arrangements that drive quality of care and promote retention (strategy 3.5)**  •Develop longer minimum service contract lengths for commissioned MH services (3.5.1).  •Identify opportunities to embed funding in service contracts to support continued professional development, and incentives (3.5.2).  **Support workplaces to create mentally healthy workplaces and adopt positive workplace cultures (strategy 3.1)**  •Develop initiatives (3.1.1) and identify opportunities to invest in infrastructure to ensure MH support is provided to the workforce (3.1.2).  •Promote the need to develop a culture of collaboration, trust, learning and accountability, and a platform where culture and values are overtly prioritised in workplace policies (3.1.3).  **Increase supervision and mentoring across all career stages, including current and emerging leaders (strategy 3.4)**  •Review guidelines for supervision and specify support requirements (3.4.1).  •Strengthen access to formal mentoring across all career stages, including future leaders (3.4.2).  **Increase access to, and use of, continuing professional development across all career stages (strategy 3.3)** •Support staff access to continuing professional development throughout their careers (3.3.1).  •Develop equitable access to continuing professional development through establishing educator roles such as in First Nations MH and LE workers (3.3.2).  •Build on existing initiatives like MHPOD to centralise details of existing training and continuing professional development opportunities available to the MH workforce on an ongoing basis (3.3.3).  **Implement evidence-driven strategies to prevent and address worker stress and burnout (strategy 3.2)**  •Adopt evidence-based practice to promote self-care and manage incidents and recovery, and implement CoP to share insights and lessons learnt (3.2.1).  •Implement training that support recognition and management of stress and burnout (3.2.2). •Strengthen processes to regularly review workloads, assess risks and develop solutions (3.2.3). | **Funding (pillar 3.2)**  •Government to commit to five yearly funding cycles, with renewal processes occurring with adequate lead time.  •Ensure competitive wages.  **Geographic distribution (pillar 2.2)**  •Government funding to incentivise regional, rural and remote pathways for the community MH workforce beyond just LE workers. This may include financial incentives to cover relocation costs,  integration supports for workers and their families, or other benefits.  **Retention (pillar 2.3)**  •Develop ongoing professional development opportunities.  •Develop leadership capability to better support workforces and lead change  •Develop clearly defined career-progressing pathways and provide appropriate support to access these opportunities.  •Government to review the NDIS pricing arrangements to ensure line items are adequate to cover service provider costs, including supervision and training of staff, and support workforce development.  **Workforce wellbeing (pillar 2.4)**  •Support the safety and wellbeing of workers through wellbeing monitoring programs and embedding additional supports to prevent fatigue, stress and burnout.  •Present the work already being done in the sector on worker wellbeing.  •Establish CoP across the sector to foster a culture of collaboration and support.  •Support organisations to implement the guidelines and principles in the National LE Workforce Development Guidelines.  •Invest in professional developmental opportunities for leaders with a focus on positive workplace culture. |
| *Wording of some strategies has been adjusted to convey more concisely. CoP: Community of Practice; MH: Mental health; LE: Lived Experience; MHPOD: Mental Health Professional Online Development. | | |
