# Supplementary material for: Identifying Solutions for the Workforce Challenges Facing Community Mental Health Support Workers: A Qualitative Study
Source: Community Ment Health J. 2025 May 22;61(7):1324–33. doi: 10.1007/s10597-025-01473-w (PMC12408665; doi:10.1007/s10597-025-01473-w)
Supplement: Supplementary file 3 — Supplementary Material 3 [file 10597_2025_1473_MOESM3_ESM.docx]

| **Supplementary Material 3.** Summary and comparison of proposed solutions for **upskilling staff**: insights from this study and two recent Australian workforce documents | | |
| --- | --- | --- |
| **Study generated solutions** | **National Mental Health Workforce Strategy 2022-2032 generated solutions*** | **Community Mental Health and Wellbeing Workforce Issues Paper*** |
| **Engaging in regular or mandatory training and supervision (subtheme 3.1)**  •Mandatory professional development sessions held every 2-3 months on topics like alcohol and drug awareness, youth work, and LGBTQIA+ support.  •PHNs to provide support in upskilling staff, rather than letting service providers handle it alone.  •New staff receive a probation document with required training for the first 6 months and a personalised development plan for ongoing growth.  •All staff receive monthly one-hour meetings with their manager as part of a formalised supervision process.  **Supporting and granting opportunities to upskill (subtheme 3.2)**  •Improving transparency about the training budget per employee and greater proactivity in following up on employees' training needs.  •If training has associated costs staff to collaborate with management to determine if it fits within the budget.  •Organisations to foster a culture where employees take charge of their own learning, identifying needed training and discussing its relevance with management.  •Organisations to support staff in completing their training (e.g. gaining a Certificate IV in MH) by allowing them the necessary time to pursue it.  **Encouraging resource sharing and networking (subtheme 3.3)**  •Staff actively share useful free online trainings, with management promoting ongoing opportunities like webinars. • (Re) Introducing local CoP to promote networking, reflection, and reduce isolation. | **Enhance training pathways, access to supervision, and support skills transfer (strategy 1.5)**  •Develop training modules that build: competencies for the provision of culturally safe, integrated and multidisciplinary care (1.5.1) and capability in suicide prevention (1.5.2).  •Increase the supply of education/training programs for both vocational and higher education trained occupations (1.5.3). •Identify opportunities to prioritise access to training for the MH workforce through increased subsidies and use of placements and traineeships (1.5.4).  •Support service providers to improve the quality and quantity of MH placements (1.5.5). •Support education providers to maintain a combination of onsite and remote education opportunities (1.5.6).  •Ensure MH students/trainees undertake placements/ internships across various settings (1.5.7).  •Support the development of basic MH skills (1.5.8).  •Review funding arrangements for education and training providers to enable delivery of micro-credentialing course work (1.5.10).  **Strengthen the capability and core competencies of the workforce to recognise and respond to community need (strategy 1.6)**  •Develop cultural safety education and training (1.6.1). •Support the MH workforce to upskill in and respond to co-occurring alcohol and other drug addiction (1.6.2) and vice-versa (1.6.3).  •Support First Nations, CALD, LGBTQIA+ and disability led initiatives to grow and develop the MH workforce to meet the needs of their communities (1.6.4).  •Support higher education providers to embed the Aboriginal and Torres Strait Islander Health Curriculum Framework into higher education curricula for a broad range of undergraduate and graduate courses and health social service career pathways (1.6.5).  **Address critical shortages in the mental health workforce (strategy 1.1)**  •Develop broader training models for the LE workforce to develop skills and capabilities (1.1.5).  •Support First Nations organisations to develop and deliver training in cultural safety, trauma informed and trauma competent care (1.1.8). •Define the competencies required by emerging and self-regulated workforces to deliver evidence-based suicide prevention, mental health promotion, treatment, support and care (1.1.9). | **Qualification development (pillar 1.1)**  a. Core qualifications  •Develop a Core Capability Framework which articulates the specific skills, knowledge, behaviours and attitudes for the MH sector which can guide course development.  •Review and enhance the content, delivery and sector perception of the current Certificate IV in Mental Health / Peer Work to ensure it aligns with industry needs.  •Develop a suite of accredited micro-credentials that will enable workers to upskill.  •Develop traineeships, in consultation with industry, to align with TAFE qualifications.  •Explore feasibility for developing new university qualifications specific to the community MH sector.  b. Leadership qualifications  •Develop a new qualification for those within the sector looking for career progression.  **Student placements (pillar 1.2)**  •Provide traineeships specific to the community MH sector.  •Provide funding for both RTOs and service providers to collaboratively deliver training and practice supervision.  •Review the length of student placements.  •Advocate for better funded placement coordinators at RTOs.  •Ongoing government funding for student mentors to coordinate student placement opportunities and offer students support.  **Professional development (pillar 1.3)**  •Establish a central ‘repository’ to coordinate learning and professional development activities across the whole sector to increase awareness of relevant training.  •Increase availability of professional development for those entering managerial / supervisory roles.  •Integrate LE expertise in the design / delivery of professional development opportunities.  •Embed professional development opportunities beyond traditional training (e.g. secondments, exchange programs, sabbaticals, coaching, mentoring).  •Fund internal workforce educators to coordinate professional development opportunities. |
| *Wording of some strategies has been adjusted to convey more concisely. CALD: Culturally and Linguistically Diverse; CoP: Community of Practice; LGBTIQ+: Lesbian, Gay, Bisexual, Transgender, Intersex, and Queer/Questioning; MH: Mental health; LE: Lived Experience; PHN: Primary Health Network; RTO: Registered Training Organisation; TAFE: Technical and Further Education. | | |
